# Analysis of potential protein-modifying variants in 9000 endometriosis patients and 150000 controls of European ancestry

**DOI:** 10.1038/s41598-017-10440-9

**Published:** 2017-09-12

**Authors:** Yadav Sapkota, Immaculata De Vivo, Valgerdur Steinthorsdottir, Amelie Fassbender, Lisa Bowdler, Julie E. Buring, Todd L. Edwards, Sarah Jones, Dorien O, Daniëlle Peterse, Kathryn M. Rexrode, Paul M. Ridker, Andrew J. Schork, Gudmar Thorleifsson, Leanne M. Wallace, iPSYCH-SSI-Broad Group, Thomas M. Werge, Wesley K. Thompson, Peter Kraft, Andrew P. Morris, Dale R. Nyholt, Digna R. Velez Edwards, Mette Nyegaard, Thomas D’Hooghe, Daniel I. Chasman, Kari Stefansson, Stacey A. Missmer, Grant W. Montgomery

**Affiliations:** 1 Department of Genetics and Computational Biology, QIMR Berghofer Medical Research Institute, Brisbane, QLD Australia; 2 Department of Epidemiology and Cancer Control, St. Jude Children’s Research Hospital, Memphis, TN USA; 3 Harvard Medical School, Boston, MA USA; 4 Channing Division of Network Medicine, Department of Medicine, Brigham and Women’s Hospital and Harvard Medical School, Boston, MA USA; 5 deCODE genetics/Amgen, Reykjavik, Iceland; 6 KULeuven, Department of Development and Regeneration, Organ systems, Leuven, Belgium; 7 Department of Obstetrics and Gynaecology, Leuven University Fertility Centre, University Hospital Leuven, Leuven, Belgium; 8 Division of Preventive Medicine, Brigham and Women’s Hospital, Boston, MA USA; 9 Institute of Medicine and Public Health, Vanderbilt University Medical Center, Nashville, TN USA; 10 Vanderbilt Genetics Institute, Division of Epidemiology, Institute of Medicine and Public Health, Department of Medicine, Vanderbilt University Medical Center, Nashville, TN USA; 11 Cognitive Science Department, University of California, San Diego, La Jolla, California USA; 12 Institute of Biological Psychiatry, Mental Health Centre Sct. Hans, Copenhagen University Hospital, DK-2100 Copenhagen, Denmark; 13 Program in Genetic Epidemiology and Statistical Genetics, Harvard T.H. Chan School of Public Health, Boston, MA USA; 14 Department of Biostatistics, University of Liverpool, Liverpool, United Kingdom; 15 Institute of Health and Biomedical Innovation, Queensland University of Technology, Queensland, Australia; 16 Vanderbilt Genetics Institute, Vanderbilt Epidemiology Center, Institute of Medicine and Public Health, Department of Obstetrics and Gynecology, Vanderbilt University Medical Center, Nashville, TN USA; 17 Department of Biomedicine, Aarhus University, Aarhus, Denmark; 18 The Lundbeck Foundation Initiative for Integrative Psychiatric Research, iPSYCH, Aarhus, Denmark; 19 Faculty of Medicine, University of Iceland, Reykjavik, Iceland; 20 Institute for Molecular Bioscience, The University of Queensland, Brisbane, QLD Australia; 21 Institute of Biological Psychiatry, Mental Health Center, Sct. Hans, Mental Health Services, Copgenhagen, Denmark; 22 The Lundbeck Foundation Initiative for Integrative Psychiatric Research, iPSYCH, Copenhagen, Denmark; 23 Institute of Clinical Sciences, Faculty of Medicine and Health Sciences, University of Copenhagen, Copenhagen, Denmark; 24 Department of Psychiatry, University of California, San Diego, La Jolla, California, United States of America

## Abstract

Genome-wide association (GWA) studies have identified 19 independent common risk loci for endometriosis. Most of the GWA variants are non-coding and the genes responsible for the association signals have not been identified. Herein, we aimed to assess the potential role of protein-modifying variants in endometriosis using exome-array genotyping in 7164 cases and 21005 controls, and a replication set of 1840 cases and 129016 controls of European ancestry. Results in the discovery sample identified significant evidence for association with coding variants in single-variant (rs1801232-*CUBN*) and gene-level (*CIITA* and *PARP4*) meta-analyses, but these did not survive replication. In the combined analysis, there was genome-wide significant evidence for rs13394619 (*P* = 2.3 × 10^−9^) in *GREB1* at 2p25.1 — a locus previously identified in a GWA meta-analysis of European and Japanese samples. Despite sufficient power, our results did not identify any protein-modifying variants (MAF > 0.01) with moderate or large effect sizes in endometriosis, although these variants may exist in non-European populations or in high-risk families. The results suggest continued discovery efforts should focus on genotyping large numbers of surgically-confirmed endometriosis cases and controls, and/or sequencing high-risk families to identify novel rare variants to provide greater insights into the molecular pathogenesis of the disease.

## Introduction

Endometriosis is a common gynecological disorder, affecting 6–10% of women of reproductive age^1^ and 20–50% of women with infertility^2^. The disease is primarily characterized by the presence of endometrial-like tissue outside the uterus, mainly due to retrograde menstrual tissue/endometrial cells attaching to pelvic structures that trigger an inflammatory response. The most common symptoms include severe pelvic pain, heavy or irregular menstrual bleeding and pain during intercourse and exercise, although some women remain asymptomatic. It is also associated with infertility, lower abdominal and back pain, diarrhoea and/or constipation and chronic fatigue. Since endometriosis affects younger working age women, there are severe economic consequences arising from loss of income, treatment costs, depression, anxiety and other disease-related disorders, posing a significant burden on patients and society^2^. Based on location, diameter and depth of lesions, and density of adhesions, there are varying degrees of endometriosis severity.

According to the revised American Fertility Society (rAFS) classification system^3^, the disease is classified into one of four (I-minimal, II-mild, III-moderate and IV-severe) stages based on location, diameter and depth of lesions, and density of adhesions. Risk factors for endometriosis include age, increased exposure to menstruation (shorter cycle length, longer duration of flow, and nulliparity) and other factors related to oestrogen levels, including decreased body mass index and smoking history^4–6^. The exact cause of endometriosis is largely unknown, but is believed to be complex, involving multiple genetic and environmental risk factors. Studies have shown that genes influence susceptibility to endometriosis and the disease has an estimated total heritability of around 0.51 from twin studies^1^ and a SNP-based heritability of 0.26^7^. We and others have conducted genome-wide association (GWA) studies for endometriosis using data from European and Japanese individuals and identified 11 independent single nucleotide polymorphism (SNP) loci at genome-wide significant level^8–12^. Subsequent meta-analysis of the European and Japanese GWA datasets further confirmed association of another locus near interleukin 1 alpha (*IL1A*) at 2q13 with endometriosis^13^. Except for rs10965235 in CDKN2B Antisense RNA 1 (*CDKN2A-AS1*) on 9p21.3, identified in the Japanese GWA study^12^, the remaining 11 SNPs are polymorphic in Europeans. We recently performed a large-scale 1000 Genomes imputation-based GWA meta-analysis including 17045 endometriosis cases and 191596 controls of both European and Japanese ancestries^14^. In addition to replicating nine out of the 11 European SNP risk loci, our results identified further ten independent SNPs. Together, the 19 endometriosis SNPs explain up to 5.19% of variance in endometriosis^14^, suggesting that many more variants remain to be identified.

GWA studies have limitations for translation and in the genome coverage of low frequency and rare variants. Most common variants associated with endometriosis risk identified from GWA studies lie in non-coding regions of the genome and detailed follow-up studies are necessary to link causal variants to altered regulation and/or function of specific genes^15^. Imputation methods have greatly improved genome coverage for common variants, but the accuracy of single variant tagging by imputation declines markedly for SNPs with minor allele frequencies (MAFs) less than 0.05^16^.

An important class of variant not well covered in our GWA studies is coding variants that modify protein composition through amino acid substitutions or altered stop signals or splicing, particularly those with MAF < 0.05. Discovery of a role for this class of variants in endometriosis would be important for future translation. Coding variants can be evaluated by whole genome or exome sequencing methods, but the cost of these methods limits large-scale studies^16^. Here, we present results from the first exome-array analysis for endometriosis, including 7164 cases and 21005 controls of European ancestry. Our specific aims were to identify novel coding variants that have not been previously captured by GWA studies as well as to assess potential role of coding variants at the previously known GWA risk loci.

## Materials and Methods

### Discovery cohorts

Six individual cohorts namely QIMR-Australia, LEUVEN-Belgium, NHS2-USA, BioVU-USA, WGHS-USA and iPSYCH-Denmark, participated in this effort representing a total sample size of 7164 endometriosis cases and 21005 controls. Full study characteristics are provided in the Supplementary Data. All endometriosis cases and approximately 70% controls in the QIMR-Australia cohort used in this study were also part of the Australian samples in our previous GWA studies^9, 10^. Of these, NHS2-USA contributed only endometriosis cases and hence were ancestrally-matched to controls from BioVU-USA, resulting in five individual exome-array case-control datasets (QIMR, LEUVEN, NHS2-BioVU, WGHS and iPSYCH) for further analysis. All studies were approved by their local institutional review boards [the QIMR Human Research Ethics Committee (QIMR), the Commission of Medical Ethics of the Leuven University Hospital (LEUVEN), the Human Subject Committee of Harvard School of Public Health and the Institutional Review Board of Brigham and Women’s Hospital (NHS2 and WGHS), the institutional review board of Vanderbilt University, and the Danish Research Ethical Committee System (iPSYCH)], and written informed consent was obtained from all study participants. All experiments were performed in accordance to the tenets of the declaration of Helsinki (7^th^ revision).

### Exome-arrays

The Illumina HumanExome, HumanCoreExome and PsychArray Beadchips are genotyping arrays containing up to 244594 exome variants, which predominantly include protein-altering variants (nonsynonymous coding, splice-site and stop gain or loss codons). Additional variants on the chip included common variants found through GWA studies, ancestry informative markers (for African and Native Americans), mitochondrial variants, randomly selected synonymous variants, HLA tag variants and Y chromosome variants. Here, we investigated association of autosomal and X-linked exome variants with endometriosis.

### Genotyping and quality control (QC)

Endometriosis cases and controls from QIMR and LEUVEN, and cases from NHS2 cohorts were genotyped at QIMR Berghofer Medical Research Institute, Brisbane, Australia. Genotyping of QIMR and NHS2 samples was performed on Illumina HumanCoreExome-12 v1.0 (~240000 exome variants) while LEUVEN samples were genotyped using Illumina HumanCoreExome-12 v1.1 (~244000 exome variants). Controls from BioVU, and cases and controls from WGHS were genotyped using Illumina HumanExome-12 v1.1 (~243000 exome variants). Cases and controls from iPSYCH samples were genotyped on Illumina Infinium PsychArray-24 v1.1 (~277000 exome variants) at the Broad Institute, USA. Genotypes were called using the GenTrain2.0 clustering algorithm within Illumina GenomeStudio software v2011.1. All studies created cluster files using their own cohort data with GenomeStudio. Genotype data were then further processed by zCall^17^, a rare variant caller to attempt to re-call missing genotypes. For iPSYCH, genotypes were called at the Broad Institute using three different algorithms: GenCall (using a predefined cluster file), Birdseed and zCall. For common SNPs (MAF > 0.01) a consensus merge between GenCall and Birdseed genotypes was done. For rare SNPs in the consensus call (MAF < 0.01), zCall genotypes passing QCs were used.

Stringent QC criteria were applied to individual exome-array case-control dataset. Briefly, samples with > 1% missingness, outlying heterozygosity, non-European ancestries based on the 1000 Genomes European populations, cryptic relatedness (pi-hat > 0.2 or GCTA relatedness cut-off > 0.08) and with gender discordance were excluded. Similarly, markers with poor separation of three genotype clusters (cluster separation score < 0.4), GenTrain score < 0.6, excess heterozygosity, outlying mean beta and intensity values for heterozygote genotypes, > 1% missing rates and with Hardy-Weinberg Equilibrium (HWE) *P* < 10^−6^ in controls were also excluded. Furthermore, variants with allele frequency significantly deviated (+/− 0.2) from those in the 1000 Genomes European population were excluded (Supplementary Figure S1).

### Single-variant association analysis

For exome variants passing the above QC, we also required minor allele count (MAC) > 3 in cases and controls separately before performing single-variant association analysis. We found this additional filter resulted in a more stable distribution of standard errors and a well-calibrated quantile-quantile (Q-Q) plots (Supplementary Figures S2–S6). Tests of association in individual datasets including all endometriosis cases and controls were performed using RareMetalWorker assuming an additive model. RareMetalWorker is a forerunner of RareMetal^18^, which produces single-variant summary statistics by fitting a linear mixed model. Genotype data for each variant were fitted as a fixed genetic effect and a genetic relationship (or kinship) matrix generated from an independent set of autosomal variants with MAF > 0.01 was included as a random effect. Samples from iPSYCH were processed in multiple batches (‘waves’) and hence a covariate was also included in the model to account for potential batch effects. In addition to the score statistics for each marker, RareMetalWorker also generates linkage disequilibrium (LD) matrices summarizing covariance between single marker statistics, which can further be utilized to perform gene-level meta-analysis without requiring individual-level genotype data. For QIMR and iPSYCH studies, which included both male and female controls, X-linked markers were analysed using female controls only.

### Single-variant meta-analysis

Since RareMetalWorker is designed for analysis of quantitative traits, we then transformed beta estimates obtained from RareMetalWorker in each dataset to a binary scale, using the intercept value from the null model^19^. For iPSYCH dataset, we used the mean trait value (i.e. the proportion of cases) as the analysis included a covariate in the linear mixed model. We then combined single-variant association results from the five case-control datasets and performed meta-analysis of all endometriosis cases versus controls, using a fixed-effect (inverse-variance weighted) model implemented in METAL^20^. Using a Bonferroni correction we declared a single variant as exome-wide significant if it reached *P* < 0.05/number of all variants reported in at least two individual case-control datasets. The variants with *P* < 1 × 10^−4^ were considered to show a suggestive association and hence were prioritized for further scrutiny.

Given the substantially greater genetic loading of moderate-to-severe (Grade B) endometriosis (rAFS III or IV disease) compared to mild or minimal (Grade A) endometriosis (rAFS I or II)^7, 10, 21^, a secondary analysis focused on suggestive variants (associated at *P* < 1 × 10^−4^), for which we performed meta-analysis of the association results from QIMR and LEUVEN Grade B cases versus controls with the NHS2-BioVU, WGHS and iPSYCH association results. As previously shown, the exclusion of minimal endometriosis cases has the potential to enrich true genetic risk effects, even taking into account the reduced sample size^7, 9, 10, 13, 21^.

Heterogeneity of allelic associations was examined using Cochran’s Q test. Between-study (effect) heterogeneity was indicated by Q statistic *P* values (*P*_*het*_) < 0.1^22^. Meta-analysis of variants associated in fixed-effect model with evidence of heterogeneity (*P*_*het*_ < 0.1) was carried out using the Han Eskin random-effects model (RE2) implemented in the METASOFT program^23^. In contrast to the conventional random-effects model, the RE2 model has greater power given heterogeneity.

Conditional analysis was performed to identify additional independent association signals at loci reaching exome-wide significance, using single-variant association summary statistics and LD matrices from individual case-control datasets using RareMetal. We then performed fixed-effect meta-analysis of the association summary statistics across the studies using the sample size Z-score weighted method in METAL. Under this method, the conditional *P* values observed in each case-control dataset were converted into a signed Z-score. We then combined Z-scores for each allele across samples in a weighted sum, with weights proportional to the square root of the sample size for each cohort. Given that our cohorts had unequal number of cases and controls, we used the effective sample size^20^, where *N*_*effective*_ = 4/(1/*N*_*cases*_ + 1/*N*_*controls*_).

### Gene-level meta-analysis

Using single-variant summary statistics and LD matrices for individual case-control datasets, gene-level meta-analysis was performed using RareMetal. In contrast to the single-variant meta-analysis, no MAC filter was imposed and hence all polymorphic variants within a study were considered. The single-variant summary statistics from all studies were then combined using the Cochran-Mantel-Haeszel method and computed variance-covariance matrices centrally^24^. We also required variants present in at least two individual studies and occur with a combined MAF < 0.05. We adapted an approach by Purcell *et al*.^25^ and focused our analyses to three primary variant sets: (i) protein truncating variants (‘Disruptive’; nonsense, frameshift and essential splice-site variants), (ii) Disruptive and non-synonymous (NS) missense variants rated as “damaging” by all of five prediction algorithms employed (PolyPhen2-HumDiv, PolyPhen2-HumVar, LRT, MutationTaster and SIFT) (‘Disruptive + NS_strict_’), and (iii) Disruptive, NS missense variants rated as “damaging” by all of five prediction algorithms employed, and NS missense variants with a combined MAF < 0.01 rated as “damaging” by at least one of five prediction algorithms employed (‘Disruptive + NS_strict_ + NS_broad(MAF < 0.01)_’). Variants were annotated using RefSeq and the annotation file from the CHARGE consortium (http://www.chargeconsortium.com/main/exomechip). We used the Combined Multivariate and Collapsing (CMC) burden test^26^ that assumes variants are unidirectional in effects, and SKAT test^27^ that is most powerful if variants have opposite directions in effect sizes.

### Replication cohort

An independent set of Icelandic endometriosis cases and controls of European origin from deCODE Genetics were used as our replication cohort^28^. Imputation of this cohort was based on whole genome sequencing of 8453 Icelanders using Illumina technology to a mean depth of at least 10X (median 32X). Approximately 30 million sequenced variants were then imputed into 150656 Icelanders who had been genotyped using Illumina genotyping arrays. Using genealogic information, the sequence variants were imputed into 294212 un-typed relatives of chip-typed individuals to further increase the sample size for association analysis and increased the power to detect associations. Of these, our replication cohort included 1840 surgically diagnosed endometriosis cases and 129016 controls, with 688 cases exhibiting moderate-to-severe (rAFS III or IV) disease. We excluded poorly imputed variants with imputation quality metric < 0.8.

### Replication and total association analyses

Association analysis of the imputed data in the deCODE replication cohort was performed as previously described^28^, and variants with a nominal *P* < 0.05 were considered to have replicated. To determine the total evidence for association, we combined single-variant association results from five individual case-control datasets with those from the deCODE, and performed a fixed-effect (inverse-variance weighted) meta-analysis for all endometriosis cases versus controls. Additional meta-analysis for QIMR and LEUVEN Grade B cases versus controls, NHS2-BioVU, WGHS and iPSYCH all cases versus controls and the deCODE Grade B cases versus controls was also performed for the variants showing suggestive association at *P* < 1 × 10^−4^ in exome-array single-variant meta-analysis of all cases versus controls.

## Results

Following quality control (QC), a total of 7164 endometriosis cases and 21005 controls from five individual case-control datasets in the discovery sample remained for the primary exome-array meta-analysis (Table 1). The number of variants passing the QC in each case-control dataset is provided in Supplementary Table S1. A total of 162203 variants passed the QC in at least two studies and were included in the gene-level analysis, and of these, 126317 were rare (MAF < 0.01), 8859 had low frequency (0.01 ≤ MAF < 0.05) and 27027 were common (MAF ≥ 0.05) in the combined discovery datasets. We restricted our single-variant analysis to 59202 variants with minor allele count (MAC) > 3 in cases and controls separately in each exome-array dataset and also present in at least two studies. Of these, 23755 were rare, 8858 had low frequency, and the remaining 26589 were common in the combined discovery datasets. Consequently, the exome-wide significance threshold for single-variant analyses was set at *P* < 8.5 × 10^−7^, corresponding to Bonferroni correction for the 59202 variants. The Q-Q plot for *P* values obtained from single-variant meta-analysis is shown in Supplementary Figure S7.

**Table 1:** Summary of the endometriosis case-control datasets (post-QC).

Dataset	Number of cases (Grade B)	Number of controls
QIMR	2223 (891)	2044
LEUVEN	998 (423)	783
NHS2-BioVU	2238	3113
WGHS	1494	14033
iPSYCH	211	1032
Exome-array meta-analysis	7164	21005
deCODE Replication	1840 (688)	129016
Total	9004	150021

### Exome variants associated with endometriosis risk

**Figure 1:**
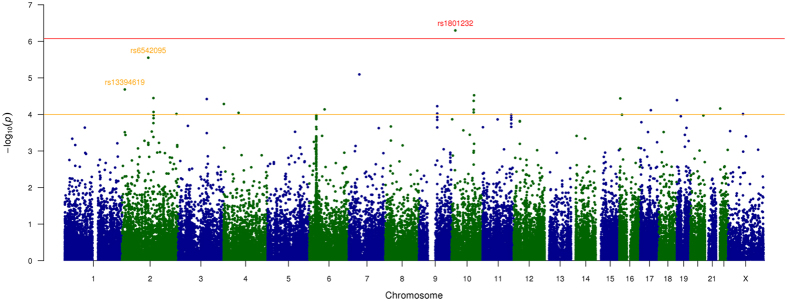
Manhattan plot of the exome-array single-variant meta-analysis results including all endometriosis cases. Red line indicates our exome-wide significance threshold (*P* = 8.5 × 10^–7^) and the orange line represents the threshold for suggestive association (*P* = 1 × 10^–4^). SNP rs1801232 (red font) on chromosome 10 achieved our exome-wide significance threshold. Two other SNPs rs6542095 and rs13394619 (light orange font) on chromosome 2 also showed strong, albeit not exome-wide significant, association in the exome-array meta-analysis of discovery cohorts. These two SNPs represent the previously identified GWA association signals at *IL1A* and *GREB1*, respectively and of these rs13394619 achieved genome-wide significance in total association analysis including all cases from both the discovery and the replication cohorts.

Single-variant meta-analysis for the five individual case-control datasets identified a non-synonymous coding variant (rs1801232) associated with endometriosis risk reaching exome-wide significance. The effect allele (T) occurring with a combined effect allele frequency (EAF) of 0.08 was associated with a decreased risk [odds ratio (OR) = 0.82; 95% confidence interval (CI) = 0.76–0.89] (Table 2 and Fig. 1). The direction of effect of the T allele was consistent across studies, except in the iPSYCH, with no statistically significant heterogeneity observed among all studies (*P*_*het*_ = 0.25) in allelic associations. Similar results (OR = 0.82; 95% CI = 0.75–0.89; *P* = 2.0 × 10^−6^; *P*_*het*_ = 0.36), although not exome-wide significant, were also observed when the meta-analysis was performed after excluding Grade A cases (Supplementary Table S2). Conditional analysis including all endometriosis cases and controls in the discovery sample did not identify further independent association signals at this locus. SNP rs1801232 is a missense variant (p.Asn3552.Lys) located in the Cubilin (*CUBN*) gene at chromosome 10p13 - a locus associated with albuminuria^29, 30^. The protein encoded by *CUBN* plays a role in lipoprotein, vitamin and iron metabolism, by facilitating their uptake.

**Table 2:** Summary of the exome-array meta-analysis and replication results for markers with *P* < 1 × 10^–4^ in analysis including ‘All’ endometriosis cases. Chr, Chromosome; Genomic position is shown relative to GRCh37 (hg19); NS, nonsynonymous; EA, effect allele; NEA, non-effect allele; EAF, average effect allele frequency in the discovery cohorts; OR, odds ratio with respect to EA; CI, confidence interval; *P*_*het*_, *P*-value from heterogeneity test; *P*_*RE2*_, *P*-value from the Han Eskin random-effects (RE2) model.

Chr	dbSNP ID	Position (bp)	Functional region	Genes	EA	NEA	Exome-array meta-analysis (all)					deCODE replication (all)		Total association analysis (all)			
							EAF	OR (95% CI)	*P*	*P*_*het*_	*P*_*RE2*_	OR (95% CI)	*P*	OR (95% CI)	*P*	*P*_*het*_	*P*_*RE2*_
10	rs1801232	16870912	NS	*CUBN*	T	G	0.08	0.82 (0.76–0.89)	7.47 × 10^–7^	0.25		0.95 (0.84–1.07)	0.37	0.86 (0.80–0.92)	4.18 × 10^−6^	0.09	5.97 × 10^–6^
2	rs6542095	113529183	intergenic	*IL1A*	T	C	0.69	0.89 (0.85–0.94)	1.71 × 10^–6^	0.75		0.93 (0.86–1.01)	0.07	0.90 (0.87–0.94)	4.39 × 10^−7^	0.76	
7	rs17172694	46437154	intergenic	*IGFBP3*	T	G	0.08	1.21 (1.11–1.30)	3.27 × 10^−6^	0.52		1.11 (0.99–1.25)	0.09	1.18 (1.10–1.26)	1.33 × 10^−6^	0.48	
16	rs78108426	11001421	NS	*CIITA*	A	C	0.01	1.87 (1.40–2.49)	1.90 × 10^−5^	0.07	2.40 × 10^−5^	0.34 (0.11–1.05)	0.06	1.68 (1.28–2.22)	2.31 × 10^−4^	0.004	6.99 × 10^−5^
10	rs138680811	98157006	NS	*TLL2*	A	G	0.003	2.44 (1.61–3.71)	2.72 × 10^−5^	0.40		1.96 (0.88–4.40)	0.10	2.33 (1.61–3.38)	7.49 × 10^−6^	0.53	
2	rs17261772	135911422	synonymous	*RAB3GAP1*	T	C	0.74	0.88 (0.83–0.94)	3.33 × 10^−5^	0.0007	1.27 × 10^−6^	1.02 (0.91–1.14)	0.78	0.91 (0.86–0.96)	4.20 × 10^−4^	0.0002	3.87 × 10^−6^
10	rs12777823	96405502	intergenic	*CYP2C18*	A	G	0.15	1.13 (1.07–1.20)	3.39 × 10^−5^	0.15		0.99 (0.89–1.11)	0.88	1.10 (1.05–1.16)	3.08 × 10^−4^	0.05	1.58 × 10^−4^
4	rs4647930	1018705	NS	*FGFRL1*	A	C	0.27	0.90 (0.86–0.95)	3.98 × 10^−5^	0.10		1.04 (0.96–1.13)	0.31	0.94 (0.90–0.98)	2.83 × 10^−3^	0.004	3.27 × 10^−4^
16	rs1639150	3747204	intronic	*TRAP1*	T	C	0.43	0.91 (0.88–0.95)	4.25 × 10^−5^	0.92		1.04 (0.97–1.12)	0.32	0.94 (0.91–0.98)	2.54 × 10^−3^	0.09	1.61 × 10^−3^
17	rs2278868	46262171	NS	*SKAP1*	T	C	0.61	1.10 (1.05–1.14)	4.38 × 10^−5^	0.35		1.07 (1.00–1.16)	0.07	1.09 (1.05–1.13)	8.73 × 10^−6^	0.45	
2	rs114242502	235951001	NS	*SH3BP4*	A	G	0.00	5.62 (2.45–12.89)	4.53 × 10^−5^	0.002	5.61 × 10^−6^	2.76 (0.07–115.4)	0.59	5.44 (2.42–12.22)	4.18 × 10^−5^	0.01	6.49 × 10^−6^
3	rs3772836	124535635	intronic	*ITGB5*	A	C	0.04	0.80 (0.72–0.89)	4.70 × 10^−5^	0.29		1.13 (0.96–1.34)	0.15	0.89 (0.81–0.97)	9.11 × 10^−3^	0.005	5.21 × 10^−4^
2	rs13394619	11727507	splicing	*GREB1*	A	G	0.48	0.92 (0.88–0.96)	5.77 × 10^−5^	0.61		0.83 (0.77–0.90)	7.43 × 10^−7^	0.89 (0.86–0.93)	2.33 × 10^−9^	0.17	
9	rs512110	79320640	NS	*PRUNE2*	T	C	0.76	1.11 (1.05–1.16)	6.61 × 10^−5^	0.71		1.04 (0.95–1.13)	0.38	1.09 (1.04–1.14)	1.04 × 10^−4^	0.59	
12	rs12313736	26383959	NS	*SSPN*	A	G	0.04	1.25 (1.12–1.39)	6.73 × 10^−5^	0.70		1.05 (0.86–1.30)	0.63	1.20 (1.09–1.32)	1.67 × 10^−4^	0.53	
6	rs9354308	66565353	intergenic	*SLC25A51P1*	A	G	0.63	0.91 (0.87–0.96)	6.88 × 10^−5^	0.94		0.92 (0.86–0.99)	0.03	0.92 (0.88–0.95)	6.69 × 10^−6^	0.98	
10	rs12767583	96547463	intronic	*CYP2C19*	T	C	0.15	1.13 (1.06–1.20)	7.07 × 10^−5^	0.54		0.98 (0.88–1.10)	0.77	1.10 (1.04–1.15)	6.97 × 10^−4^	0.19	
4	rs11131511	64933220	intergenic	*LPHN3-AS1*	T	G	0.54	1.09 (1.05–1.14)	7.24 × 10^−5^	0.30		0.95 (0.89–1.03)	0.21	1.05 (1.02–1.09)	6.08 × 10^−3^	0.01	1.08 × 10^−3^
2	rs1446585	136407479	intronic	*R3HDM1*	A	G	0.70	0.89 (0.84–0.94)	8.08 × 10^−5^	0.001	4.63 × 10^−6^	0.98 (0.89–1.09)	0.73	0.91 (0.87–0.96)	3.04 × 10^−4^	0.001	1.13 × 10^−5^
12	rs12313670	26383834	NS	*SSPN*	A	G	0.04	1.24 (1.12–1.38)	8.42 × 10^−5^	0.75		1.05 (0.85–1.30)	0.63	1.20 (1.09–1.32)	1.96 × 10^−4^	0.59	
10	rs1126545	96493058	NS	*CYP2C18*	T	C	0.15	1.13 (1.06–1.19)	8.51 × 10^−5^	0.60		0.98 (0.88–1.10)	0.76	1.09 (1.04–1.15)	8.53 × 10^−4^	0.21	
22	rs148613283	18301508	NS	*MICAL3*	T	C	0.99	1.68 (1.29–2.17)	9.04 × 10^−5^	0.40		1.17 (0.68–2.01)	0.58	1.57 (1.24–1.98)	1.61 × 10^−4^	0.37

Our primary exome-array meta-analysis including all cases also identified a further 21 variants showing suggestive (*P* < 1 × 10^−4^) association with endometriosis risk (Table 2). Most (n = 12) in the list are coding variants, with 11 non-synonymous and one synonymous variant, and of these five are low frequency coding variants. Interestingly, two (rs6542095 and rs13394619) out the nine non-coding SNPs in the list, are located in genes previously associated GWA risk loci, both reported by ourselves^9, 13^. The intergenic SNP rs6542095 near *IL1A* is ranked second in the list of 22 variants, and the effect allele T is strongly associated with decreased risk (OR = 0.89; 95% CI = 0.85–0.94; *P* = 1.7 × 10^−6^; *P*_*het*_ = 0.75) of endometriosis (Table 2), with slightly stronger association (OR = 0.88; 95% CI = 0.84–0.93; *P* = 8.9 × 10^−7^; *P*_*het*_ = 0.56) with Grade B endometriosis (Supplementary Table S2). SNP rs13394619 is intronic to the growth regulation by estrogen in breast cancer 1 gene (*GREB1*) where it disrupts a splice site, and the effect allele A is also associated with decreased risk (OR = 0.92; 95% CI = 0.88–0.96; *P* = 5.8 × 10^−5^; *P*_*het*_ = 0.61) of endometriosis (Table 2), with similar results (OR = 0.92; 95% CI = 0.88–0.96; *P* = 4.6 × 10^−4^; *P*_*het*_ = 0.69) in Grade B analysis (Supplementary Table S2). Association results of all the 22 variants in the individual exome-array dataset are provided in Supplementary Table S3.

In our replication analysis, rs1801232 with exome-wide significance did not replicate (*P* = 0.37) in an independent set of endometriosis cases and controls from the deCODE Genetics cohort. Results including Grade B cases in the deCODE cohort (OR = 0.99; *P* = 0.95) were also not significant. Consequently, rs1801232 did not retain exome-wide significance in the total association analyses (‘All’: *P* = 4.2 × 10^−6^, ‘Grade_B’: *P* = 1.6 × 10^−5^) after combining results from both discovery and replication cohorts (Table 2 and Supplementary Table S2). The association strength did not increase (*P* = 5.9 × 10^−6^) even after accounting for the observed heterogeneity through an all endometriosis total association analysis (*P*_*het*_ = 0.09) in the RE2 model.

Of the remaining 21 variants with *P* < 1 × 10^−4^ in the exome-array single-variant meta-analysis including all endometriosis cases and controls, two intergenic common SNPs (rs13394619 and rs9354308) survived independent replication (*P* < 0.05) in the deCODE cohort including all endometriosis cases, with the same direction of effect to those observed in the discovery cohorts (Table 2 and Supplementary Table S3). SNP rs13394619 is the *GREB1* GWA signal that showed associations with both all (*P* = 7.4 × 10^−7^) (Table 2) and Grade B (*P* = 0.032) (Supplementary Table S2) endometriosis. The other intergenic SNP rs9354308 near solute carrier family 25 member 51 pseudogene 1 (*SLC*2*5A51P1*) also survived independent replication in analysis including all (*P* = 0.03) but not Grade B (*P* = 0.42) endometriosis cases from deCODE cohort.

**Figure 2:**
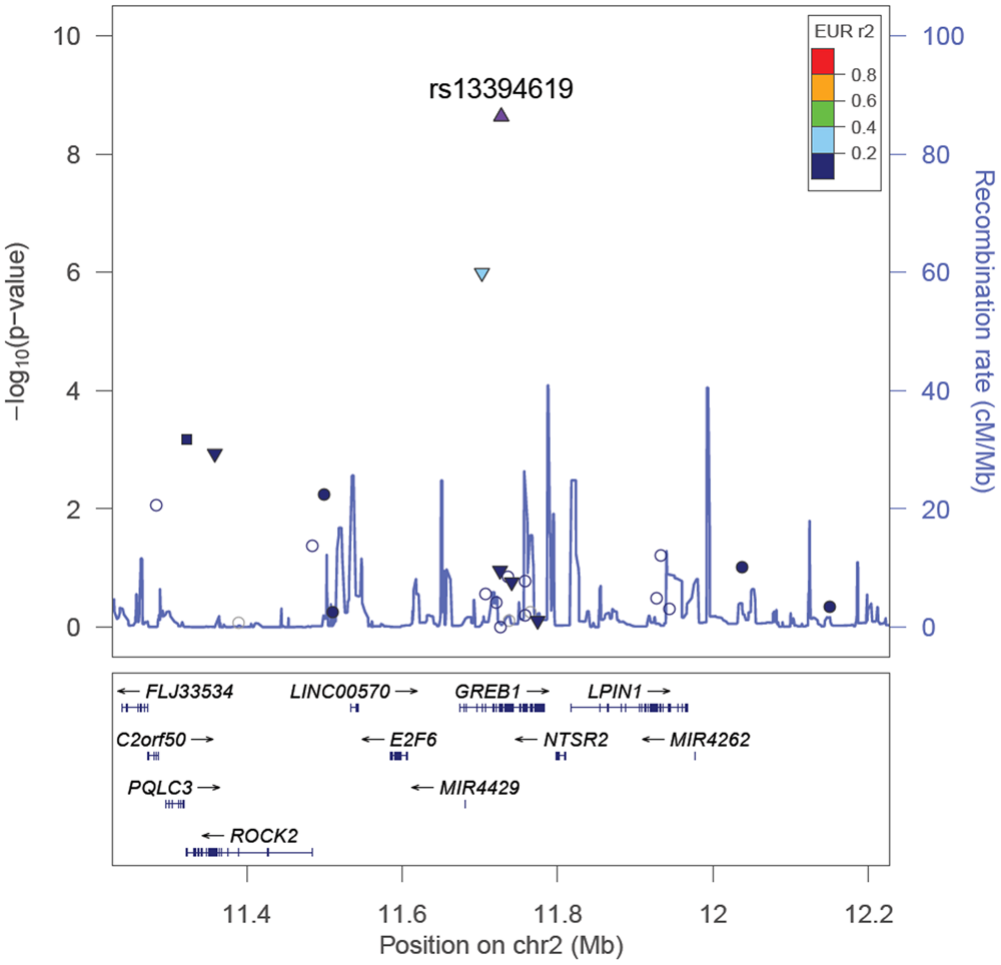
Evidence of association with all endometriosis from the total association analysis across the 2p25.1 (*GREB1*) region. SNPs are shown as circles, triangles or squares, and different shapes denote the different categories of the SNPs: up-triangle for frameshift or splice SNPs, down-triangle for nonsynonymous SNPs, square for coding or untranslated region (UTR) SNPs; star for SNPs in tfbscons region (in a conserved region predicted to be a transcription factor binding site), square filled with ‘X’ symbol for SNPs located in mcs44placental region (in a region highly conserved in placental mammals) and circle for SNPs with no annotation information. The lead SNP at each known GWA risk locus reported by Nyholt *et al*.^9^, if present in our exome-array single-variant meta-analysis, is represented by a purple triangle. All other SNPs are color coded according to the strength of LD with the top genotyped SNP (as measured by *r* ^*2*^ in the European 1000 Genomes data).

In the total association analysis including results from both the discovery and replication cohorts, the *GREB1* GWA signal achieved genome-wide significance (OR = 0.89; 95% CI = 0.86–0.93; *P* = 2.3 × 10^−9^; *P*_*het*_ = 0.17) with all endometriosis cases (Table 2 and Fig. 2). The association signal persisted (*P* = 5.3 × 10^−5^; *P*_*het*_ = 0.73), albeit not genome-wide significant, in analysis including Grade B cases (Supplementary Table S2). Similarly, the *IL1A* GWA signal showed a near genome-wide significant (*P* = 4.4 × 10^−7^; *P*_*het*_ = 0.76) association with all (Table 2) endometriosis and the signal was comparable for Grade B (*P* = 5.1 × 10^−7^; *P*_*het*_ = 0.65) cases (Supplementary Table S2), with no evidence of heterogeneity in allelic associations (*P*_*het*_ = 0.55).

### Genes associated with endometriosis risk

We also carried out gene-level meta-analysis using burden and SKAT tests for the three alternate variant sets, as described in Methods. Gene-based tests take into account overall variant-load within a specific gene/locus and therefore may have greater power than single-variant tests to detect multiple rare and low frequency alleles. We tested a total of 755, 3635 and 12373 genes with at least two variants in ‘Disruptive’, ‘Disruptive + NS_strict_’ and ‘Disruptive + NS_strict_ + NS_broad(MAF < 0.01)_’ categories, respectively. We declared a gene-based association as exome-wide significant at *P* < 1.5 × 10^−6^, corresponding to Bonferroni correction for the 16763 genes each for burden and SKAT tests. For gene-based tests reaching exome-wide significance, if the signal was driven by a single variant, we also required the variant to achieve exome-wide significance in the single-variant test.

Results from gene-based meta-analysis identified class II major histocompatibility complex transactivator (*CIITA*) and poly(ADP-ribose) polymerase family member 4 (*PARP4*) as associated with endometriosis risk at exome-wide significance level (Supplementary Table S5). *CIITA* showed exome-wide significant association in both burden (*P* = 5.9 × 10^−7^) and SKAT (*P* < 1.5 × 10^−9^) tests (Supplementary Tables S4 and S5), and the gene included eight non-synonymous coding variants with an average combined MAF of 0.0008. The observed *CITTA* genic signal included eight variants and the association was primarily driven by three variants (rs144646271, rs78108426 and rs113889330) with single-variant meta-analysis *P* values as 0.003, 9.1 × 10^−6^, 0.08, respectively (Supplementary Tables S4 and S5). We could not examine the genic association of *CIITA* in our independent deCODE cohort because directly measured genotype data were not available. However, results from single-variant analyses including both all and Grade B cases in deCODE indicated borderline association for rs78108426 (*P* < 0.06 and 0.05, respectively), but the direction of effect was opposite to the discovery cohorts. SNP rs144646271 showed *P* values of 0.28 and 0.50 in all and Grade B cases, respectively, and data for rs113889330 was not available in the deCODE cohort.

*PARP4* also showed exome-wide significant association with endometriosis risk in the SKAT test (*P* = 5.9 × 10^−7^). The *PARP4* genic signal was also strong in the burden test (*P* = 1.4 × 10^−5^) (Supplementary Table S4). While *PARP4* included two splicing variants (rs201251739 and rs142749081) with an average combined MAF of 0.0002, the observed genic association signal in SKAT test was primarily driven by rs201251739 with a single-variant meta-analysis *P* = 3.2 × 10^−7^. However, considering 158914 polymorphic autosomal variants in at least two individual studies used to produce gene-level meta-analysis results, rs201251739 does not reach the exome-wide significance level in the corresponding single-variant test. Notably, rs201251739 does not replicate in both all (*P* = 0.87) and Grade B (*P* = 0.91) deCODE cohort. As such, the genic signal for *PARP4* observed in SKAT could not be considered to achieve the exome-wide significance. Other genes observed in the list of top five hits in each of the three variant (‘Disruptive’, ‘Disruptive + NS_strict_’ and ‘Disruptive + NS_strict_ + NS_broad(MAF < 0.01)_’) categories from both burden and SKAT tests (*P* < 1.1 × 10^−3^) are provided in Supplementary Tables S4 and S5, respectively).

## Discussion

In this study we genotyped endometriosis cases and controls using Illumina genotyping arrays containing the Human Exome content with 244594 variants. The coding variants can change protein function and any such variants associated with endometriosis would highlight genes directly responsible for increased disease risk. The majority of variants are rare or occur with a low frequency and hence may not be well imputed in GWA studies^16^. This study, including data from 9004 endometriosis cases and 150021 controls of European ancestry, represents the first large discovery effort to search for association between exome variants and endometriosis and provides important insights on their potential impact in endometriosis pathogenesis.

Following single variant analysis of 59202 variants passing our quality control on the Illumina exome-arrays, there was one missense variant (rs1801232; p.Asn3552.Lys) in *CUBN* that reached exome-wide significance level. Our gene-based analyses of exome-array data identified *CIITA* and *PARP4* as significantly associated with endometriosis susceptibility. The *CIITA* genic signal was primarily driven by three missense variants that also showed significant associations in single-variant meta-analysis, lending support to the observed gene-based signal. The *PARP4* signal was mainly driven by a single variant. However, the association with rs1801232 in *CUBN* and the *CIITA* and *PARP4* genic signals could not be replicated in an independent dataset. Neither *CIITA* nor *PARP4* showed association with endometriosis in the GWA gene-based analyses^14^, although *CUBN* was nominally significant (*P* = 1.35 × 10^−3^).

Exome-arrays provide a fast and economical platform to investigate the role of coding variants, but have some limitations^31^. Whole genome or exome sequencing would provide a more comprehensive evaluation of coding variants, including novel variants, copy number variants, and structural variation. However, the cost of exome sequencing remains high limiting use for large-scale studies with sufficient power to detect association with low-frequency variants. The variants included in the exome-arrays are estimated to represent 97–98% of the non-synonymous, and 94–95% of the splice altering and stop altering variants detected in an average genome through exome sequencing (http://genome.sph.umich.edu/wiki/Exome_Chip_Design#Online_Content). Our discovery sample size had ~93% and ~89% power to detect exome variants of frequency 0.05 and 0.01 contributing to genotype relative risk of 1.30 and 1.65, respectively^32^, and if low frequency coding variants with moderate effect size contributed to endometriosis risk they would have been observed in this study. We cannot rule out the possibility that low frequency coding variants with moderate or large effects exist in non-Europeans or severely affected families, but our results did not identify any coding variants (MAF > 0.01) with moderate or large effect sizes influencing endometriosis risk.

Results from previous studies have shown that moderate-to-severe endometriosis cases have greater genetic loading than minimal or mild disease^9, 10, 21^. Only about 18% of endometriosis cases in our discovery sample had moderate-to-severe disease and hence this study may not have adequately examined the potential impact of coding variants in severe cases. Moreover, 77% (gene-based analysis) and 40% (single-variant tests) of the exome variants examined in this study are rare (MAF < 0.01), and our study had limited power to detect association of such variants with endometriosis. Additionally, very rare variants (MAF < 0.001) are not well captured by this exome-array based approach. Whole exome sequencing in reasonably large samples, possibly tens of thousands of cases and similar number of controls, or high-risk families will be required to fully understand the role for rare coding variation in endometriosis pathogenesis. However, given a fixed budget and the relative costs of genotyping arrays and whole genome sequencing, genotyping and imputation of a much larger sample will have greater power to detect both common and low frequency variants^16^. The large reference panel now available for imputation further increases the accuracy of imputation of low frequency and rare variants^33^.

Results from the analysis of both discovery and replication samples did provide support for near genome-wide significant association for rs6542095 near *IL1A* and significant association of the splice variant rs13394619 at the *GREB1* locus with endometriosis. SNP rs6542095 is intergenic, located ~34 kb distant and in very strong LD (*r* ^2^ = 0.96) with rs10167914, the most strongly associated SNP at this locus in a recent study of common variants associated with endometriosis^14^. The *GREB1* signal was first identified by ourselves^34^ in the GWA meta-analysis comprising both European and Japanese individuals. In the current study of European samples, results for rs13394619 were genome-wide significant (*P* = 2.3 × 10^−9^), although this includes the Australian samples (all endometriosis cases and approximately 70% of the controls) from our previous GWA meta-analysis. The effect size was similar, and the direction was consistent with previous reports and exclusion of the Australian samples provided comparable results (OR = 0.89 and *P* = 5.8 × 10^−8^). *GREB1* is an excellent candidate in endometriosis pathogenesis because of its known estrogen-responsive properties and previous reports of altered *GREB1* expression both at mRNA and protein levels^35^. Recent meta-analysis of common variants associated with endometriosis^14^ identified two independent signals at the *GREB1* locus, rs77294520 located approximately 13 kb upstream of *GREB1* and rs11674184 located in intron 7 of *GREB1*. The SNP rs13394619 is a splice acceptor variant located approximately 6 kb from rs11674184 and the two SNPs are in strong LD (*r* ^*2*^ = 0.66) based on the 1000 Genomes Europeans data. The Ensembl database^36^ reports 10 transcripts for *GREB1* producing 8 different proteins and effects of rs13394619 on specific *GREB1* transcripts should be evaluated in RNA sequence data for endometrium.

There was no evidence that coding variants in other relevant genes can account for the association of previously identified GWA risk loci with endometriosis (Supplementary Figures S8–S14). A number of candidate gene studies tested association with coding variants, although many had issues with study design and very few results have been replicated^37, 38^. Our results did not identify association with coding variants in any of the candidate genes including genes involved in steroid hormone synthesis and response (aromatase (*CYP19A1*), estrogen receptor 1 (*ESR1*), estrogen receptor 2 (*ESR2*), or progesterone receptor (*PGR*)), or strong independent association in genes within regions identified from GWA studies. The most recent GWA meta-analysis including 17045 endometriosis cases and 191596 controls of both European and Japanese ancestries^14^ identified 19 independent signals in 14 genomic regions associated with endometriosis, which together explain 1.75% and 5.19% of variance in all and moderate-to-severe endometriosis^14^. In addition to *GREB1*, the meta-analysis identified risk variants located near genes for ESR1 and follicle stimulating hormone beta subunit (*FSHB*). Lack of evidence for coding variants associated with endometriosis in these or other genes within regions identified from GWA studies suggests common causal variants associated with disease act through functional changes in regulatory sequences in these regions.

In summary, we conducted the first large exome-array analysis and evaluated the potential role of coding variants in endometriosis risk. Despite sufficient power, our results did not identify any coding variants (MAF > 0.01) with moderate or large effect sizes in endometriosis pathogenesis, although we will have missed variants not assayed on the Illumina exome-arrays. Only ~23% of the exome variants examined in this study occur with MAF > 0.01 and our results do not exclude effects of rare variants in other populations or high risk families. Our results did not identify association with coding variants in most putative candidate genes, but do provide additional evidence for genome-wide significant evidence for association with a splice variant in the *GREB1* locus in women with European ancestry. We conclude that continuing gene discovery efforts in endometriosis should focus on genotyping large numbers of surgically confirmed endometriosis cases and controls. In addition, sequencing high-risk families may identify novel rare variants that could provide further insights into the molecular pathogenesis of the disease.

### Data availability

The authors declare that the data supporting the findings of this study are available within the article and its supplementary information files. For additional data (beyond those included in the main text and Supplementary Information) that support the findings of this study, please contact the corresponding author.

## Electronic supplementary material

Supplementary Information

Supplementary Table 1

Supplementary Table 2

Supplementary Table 3

Supplementary Table 4

Supplementary Table 5
