# Supplementary material for: Analysis of potential protein-modifying variants in 9000 endometriosis patients and 150000 controls of European ancestry
Source: Sci Rep. 2017 Sep 12;7:11380. doi: 10.1038/s41598-017-10440-9 (PMC5595920; doi:10.1038/s41598-017-10440-9)
Supplement: Supplementary file 1 — Supplementary Information [file 41598_2017_10440_MOESM1_ESM.pdf]

Analysis of potential protein-modifying variants in 9000 endometriosis patients and 150000 controls of European ancestry

Yadav Sapkota[1,2,*], Immaculata De Vivo[3,4], Valgerdur Steinthorsdottir[5], Amelie Fassbender[6,7], Lisa Bowdler[1], Julie E. Buring[3,8], Todd L Edwards[9], Sarah Jones[10], Dorien O[6,7], Daniëlle Peterse[6,7], Kathryn M. Rexrode[3,8], Paul M Ridker[3,8], Andrew J Schork[11,12], Gudmar Thorleifsson[5], Leanne M Wallace[1], iPSYCH-SSI-Broad Group, Peter Kraft[13], Andrew P Morris[14], Dale R Nyholt[1,15], Digna R Velez Edwards[16], Mette Nyegaard[17,18], Thomas D'Hooghe[6,7,19], Daniel I Chasman[3,8], Kari Stefansson[5,20], Stacey A Missmer[3,4] and Grant W Montgomery[1,21]

Discovery Cohorts

QIMR

In total, 4,312 individuals, consisting of 2,232 endometriosis patients were recruited by The Queensland Institute of Medical Research (QIMR), Brisbane, Australia, between 1995-2002 (each completing a questionnaire and providing a blood sample). Surgical diagnosis for all endometriosis cases was confirmed from retrospective examination of medical records[1]. The QIMR case sample consisted of one case per family from families with affected sister pairs and other affected relatives, a case from each of triad families (one affected plus her parents) and further cases with no ascertained family members. QIMR controls were a sample of 2,080 parents and siblings of adolescent twins recruited as part of the Brisbane Adolescent Twin Study[2,3], where adolescent twins, their siblings and parents were recruited through schools around Brisbane, Australia. Approval to obtain medical records, for collection of blood for DNA extraction and all questionnaires and interview schedules, and for the inclusion of some twins from the Australian Twin Registry, was obtained from the QIMR Human Research Ethics Committee (HREC). All participants gave written informed consent.

Staging of disease in QIMR cases was done based on the revised American Fertility Society (rAFS) classification system[4]. Disease severity was assessed retrospectively from medical records by use of rAFS classification system, which assigns patients to one of four stages (I-IV) on the basis of the extent of disease and the associated adhesions present.

LEUVEN

Endometriosis cases (n = 1,077) and controls (n = 900) included in this cohort were recruited at the Leuven University Hospital, Belgium during 1993 - 2012, who had undergone laparoscopy for subfertility with or without pain[5]. Presence of endometriosis in cases was laparoscopically- and histologically-confirmed based on electronic medical file records. The disease severity in women with endometriosis was prospectively graded according to the rAFS classification system [4]. Absence of endometriosis in controls was confirmed laparoscopically. Both cases and controls were Caucasian in origin. All the study participants provided written informed consent, and the study was

approved by the Commission of Medical Ethics of the Leuven University Hospital, Belgium.

NHS2

NHS2 endometriosis cases were drawn from US Nurses' Health Study (NHS) II[6,7], a prospective cohort study with follow-up from 1989-2007. Biennially, 116,678 registered female nurses – aged 25-53 and residing in 14 of the US states – complete questionnaire information on incidence of disease outcomes and biological, environmental, dietary, and life-style risk factors. From 1996-1998, blood samples were collected from 29,613 participants 32-53 years of age. Women were asked if they had "ever had physician-diagnosed endometriosis", the date of diagnosis, and whether diagnosis had been confirmed by laparoscopy. To assess the validity of self-reported endometriosis, the laparoscopy records of 200 randomly selected women who had reported a diagnosis from 1989-1993 were sought; the diagnosis was confirmed in 96% of 105 women who had surgery and whose records were available[6,7]. The US case dataset comprised 2,540 cases with a self-reported laparoscopy-confirmed diagnosis of endometriosis and available blood samples, all of self-reported European descent.

BioVU

The BioVU Repository (2007 – present) is located at Vanderbilt University, Nashville, TN and was designed to link clinical data available from de-identified electronic medical records to DNA specimens[8]. The BioVU Repository consists of de-identified blood samples obtained from patients at Vanderbilt University Medical Center Hospital, including all clinics that are part of the hospital system. De-identified data from multiple sources are available within BioVU, including diagnostic and procedure codes, basic demographics, discharge summaries, clinical notes and reports, health history, laboratory values, electronically derived data, and inpatient medication orders. BioVU controls (n = 3,113) were females between the ages of 18 and 65, and were constructed two ways: 1) our primary control definition will be based on including eligible women 18 to 65 whom have no reported diagnosis (ICD9) or text mention of endometriosis; 2) the second set consisted of women 18 to 65 that have had a laparoscopic procedure where endometriosis would have been discovered were it present. This second set was

used to conduct sensitivity analyses to assess the quality of our control definition. This study of de-identified data was determined to be non-human subject research by the institutional review board (IRB) of Vanderbilt University, Nashville, TN. All BioVU controls were of Caucasian origin based on self-reported questionnaire.

WGHS

The Women's Genome Health Study (WGHS)[9] is a prospective cohort of initially healthy, female North American health care professionals at least 45 years old at baseline representing participants in the Women's Health Study (WHS) who provided a blood sample at baseline and consent for blood-based analyses. The WHS was a 2 × 2 trial beginning in 1992-1994 of vitamin E and low dose aspirin in prevention of cancer and cardiovascular disease with about ten years of follow-up. Since the end of the trial, follow-up has continued in observational mode. Additional information related to health and lifestyle were collected by questionnaire throughout the WHS trial and continuing observational follow-up.

Endometriosis status was ascertained by the eighth questionnaire during the observational follow-up. WHS participants were asked: "Have you EVER had physician-diagnosed endometriosis (yes/no)? If yes, has your endometriosis diagnosis been confirmed by laparoscopy (a standard method for diagnosing endometriosis) (yes/no/unsure)?" From these questions, endometriosis status was defined two ways. A stringent definition specified cases who responded "yes" to both questions. All other endometriosis cases were defined by response "yes" to the first question, and a response of "no", "unsure", or "missing" to the second question. Controls were defined as responding "no" to the first question. These controls were equally divided at random into two groups for pairing with the two sets of cases such that no participants were shared by analyses of two case definitions.

iPSYCH

Endometriosis cases (n = 212) and age-matched controls (n = 1032) were drawn from the iPSYCH sample, a new population-based sample of more than 80,000 samples derived from the Danish Neonatal Screening Biobank. In short the Danish Neonatal Screening Biobank comprise dried bloodspots (Guthrie cards) from all individuals born

in Denmark since 1981, where DNA extracted from the bloodspots can be successfully amplified and employed in GWAS[10]. All samples can be linked to the Danish register system, including the Danish National Patient Register (DNPR) that contains information about all hospital admissions and discharge, diagnoses and surgical procedures since 1977[11]. The 80,000 iPSYCH samples were drawn primarily to study mental disorders, including approximately 30,000 population-based controls. The endometriosis cases were identified from the entire iPSYCH sample using ICD-8: 625.30–39 and ICD-10: N80.0–N80.9 (the 9th revision of ICD was not used in Denmark) from the DNPR. The control individuals were selected to be age-matched individuals from the iPSYCH controls. All endometriosis cases in the iPSYCH sample were younger than 32 years. This study has been approved by the Danish research ethical committee system.

References

1. Treloar, S.A. *et al.* Genomewide linkage study in 1,176 affected sister pair families identifies a significant susceptibility locus for endometriosis on chromosome 10q26. *Am J Hum Genet* **77**, 365-76 (2005).

2. McGregor, B. *et al.* Genetic and environmental contributions to size, color, shape, and other characteristics of melanocytic naevi in a sample of adolescent twins. *Genet Epidemiol* **16**, 40-53 (1999).

3. Zhu, G. *et al.* A major quantitative-trait locus for mole density is linked to the familial melanoma gene CDKN2A: a maximum-likelihood combined linkage and association analysis in twins and their sibs. *Am J Hum Genet* **65**, 483-92 (1999).

4. American Fertility Society. Revised American Fertility Society classification of endometriosis: 1985. *Fertil Steril* **43**, 351-2 (1985).

5. Sundqvist, J. *et al.* Replication of endometriosis-associated single-nucleotide polymorphisms from genome-wide association studies in a Caucasian population. *Hum Reprod* **28**, 835-9 (2013).

6. Missmer, S.A. *et al.* Incidence of laparoscopically confirmed endometriosis by demographic, anthropometric, and lifestyle factors. *Am J Epidemiol* **160**, 784-96 (2004).

7. Vitonis, A.F., Baer, H.J., Hankinson, S.E., Laufer, M.R. & Missmer, S.A. A prospective study of body size during childhood and early adulthood and the incidence of endometriosis. *Hum Reprod* **25**, 1325-34 (2010).

8. Pulley, J., Clayton, E., Bernard, G.R., Roden, D.M. & Masys, D.R. Principles of human subjects protections applied in an opt-out, de-identified biobank. *Clin Transl Sci* **3**, 42-8 (2010).

9. Ridker, P.M. *et al.* Rationale, design, and methodology of the Women's Genome Health Study: a genome-wide association study of more than 25,000 initially healthy american women. *Clin Chem* **54**, 249-55 (2008).

10. Hollegaard, M.V. *et al.* Robustness of genome-wide scanning using archived dried blood spot samples as a DNA source. *BMC Genet* **12**, 58 (2011).

11. Lynge, E., Sandegaard, J.L. & Rebolj, M. The Danish National Patient Register. *Scand J Public Health* **39**, 30-3 (2011).
